# Chitosan–Zein Films Decorated with Green Synthesized Silver Nanoparticles Using *Bergenia ciliata* Extract

**DOI:** 10.3390/molecules30112311

**Published:** 2025-05-24

**Authors:** Ananda Bahadur Chand, Moses Ashie, Rabin Dahal, Ram Datt Joshi, Arjun Prasad Tiwari, Mahesh Kumar Joshi, Bishnu Prasad Bastakoti, Surya Kant Kalauni

**Affiliations:** 1 Central Department of Chemistry, Tribhuvan University, Kirtipur 44618, Nepal; ananda@bostoncollege.edu.np (A.B.C.); rdjoshi88@gmail.com (R.D.J.); mahesh.joshi@trc.tu.edu.np (M.K.J.); 2 Department of Chemistry, Patan Multiple Campus, Tribhuvan University, Kirtipur 44618, Nepal; 3 Department of Chemistry, North Carolina A&T State University, Greensboro, NC 27411, USA; mdashie@aggies.ncat.edu (M.A.); rdahal@aggies.ncat.edu (R.D.); 4 Department of Mechanical Engineering and Engineering Science, University of North Carolina at Charlotte, Charlotte, NC 28223, USA; tiwariarjuna@gmail.com

**Keywords:** antimicrobial, silver nanoparticles, chitosan, zein, capping properties

## Abstract

This study reports on the fabrication of chitosan-zein films decorated with silver nanoparticles synthesized via a green and eco-friendly approach using an extract of *Bergenia ciliata*. The strong reducing ability and caping properties of the phytochemicals present in the extract enabled the formation of silver nanoparticles with an average diameter of ~40 nm without any external reducing agent and stabilizer. The uniformly distributed Ag nanoparticles were embedded in the chitosan-zein matrix. The structural and morphological characteristics of the film were analyzed via Fourier transform infrared spectroscopy, X-ray photoelectron spectroscopy, X-ray diffractometry, scanning electron microscopy, and transmission electron microscopy. The silver nanoparticles decorated with chitosan-zein films showed thermal stability, durability, and excellent antimicrobial activities. This finding demonstrates a sustainable and green pathway for developing multifunctional nanocomposite films, contributing to the advancement of bio-based materials for prospective therapeutic applications and active food packaging.

## 1. Introduction

Silver nanoparticles (AgNPs) possess exceptional physicochemical properties, making them valuable in diverse industries, including materials engineering, biosensing, pharmaceuticals, healthcare, food packaging, water treatment, and consumer products. The unique properties of AgNPs such as thermal stability, optical and electrical conductivity, antimicrobial, antiviral, and catalytic activities, contribute to their widespread applications [1,2,3]. However, AgNPs are prone to agglomeration, affecting their stability, bioavailability, and toxicity, highlighting the need for enhanced performance strategies. The optical properties of these materials have also been shown to interfere with their degree of toxicity [4,5]. Various approaches, including polymeric matrices and functionalized supports, have been explored to improve the stability and functionality of AgNPs. Anchoring nanoparticles in a film or polymer will minimize agglomeration, limit exposure to toxicity, and provide additional support for nanoparticles [6,7]. AgNPs synthesized from plant extracts exhibit antioxidant and antimicrobial properties and the ability to control particle shape and size, making them suitable for biomedical and packaging applications [8,9]. Ghasemi et al. synthesized the AgNPs and chitosan-coated silver nanoparticles (CS-AgNPs) utilizing a methanolic extract of the *Ferula gummosa Boiss.* Gum is a green-reducing and stabilizing agent [10]. Similarly, Liu et al., synthesized AgNPs using extracts of *Eriobotrya japonica* and populus leaves, emphasizing the crucial role of flavonoids in reducing Ag^+^ to Ag^0^ and their antibacterial application [11].

In parallel, developing biodegradable and biocompatible materials for packaging and medical applications has gained increasing attention. Zein, a prolamin protein derived from corn, has shown significant potential as a biodegradable and biocompatible material for food and pharmaceutical packaging. However, its hydrophobic nature and mechanical limitations restrict its standalone application [12]. To overcome these drawbacks, researchers have incorporated plasticizers, crosslinkers, and bioactive additives to enhance their flexibility and tensile strength, but they exhibit poor moisture resistance in humid conditions [12,13]. Recently, zein‒chitosan composites have emerged as highly applicable for numerous applications by varying the ratio of zein to chitosan, modifying the thermal properties, fabricating composite materials with varying thicknesses, adding different components with well-defined properties, and applying many emerging research technologies. Pavlátková et al. synthesized bioactive zein-chitosan composite materials decorated with essential oils for application in food packaging [14]. This modification creates an antimicrobial environment for packaged products, extending their shelf-life. The formation of layered zein-chitosan-zein sandwich-like nanofibers modified with teicoplanin as a local antibacterial agent was found to be very effective for drug delivery [15]. Edible films with antioxidants and antimicrobial properties were fabricated via the incorporation of dicarboxylic acids and phenolic compounds into zein-chitosan composite films [16]. Other modifications have focused on thermal stability, tensile properties, and durability. Thermal stability has been identified as an essential factor in food packaging techniques that require materials that are resistant to heat and other thermal conditions [17,18].

Despite the growing interest in biopolymer-based nanocomposites, limited research has explored the integration of AgNPs synthesized from medicinal plant extracts into chitosan-zein matrices. While various AgNP-biopolymer composites have been studied, incorporating plant extract-derived nanoparticles to enhance zein-chitosan matrices’ physicochemical, antimicrobial, and mechanical properties remains underexplored. Most studies on AgNP-polymer composites focus on synthetic methods or commonly used plant extracts or synthetic polymers, while the potential of *Bergenia ciliata* (BC) in nanoparticle synthesis remains largely unexplored. Additionally, the synergistic effects of BC-derived AgNPs with chitosan and zein for improving antimicrobial activity, thermal stability, and film durability have not been systematically investigated. Furthermore, there is a lack of research on the synthesis of AgNPs using BC extract, a medicinal plant native to the Himalayan region of Dolpa, Nepal. It is traditionally known for its antimicrobial and antioxidant properties, making it a promising candidate for green nanoparticle synthesis. This study explores the green synthesis of AgNPs using BC extract from the Himalayan region of Nepal and their integration into a chitosan-zein biopolymer matrix to develop a biodegradable, antimicrobial, and thermally stable material. The natural bioactive compounds present in BC contribute antimicrobial and antioxidant properties, enhancing the overall functionality of the composite. The least-explored combination of BC-derived AgNPs with chitosan-zein offers a sustainable alternative for food packaging, drug delivery, and wound healing applications. This research advances the development of environmentally friendly nanocomposites by utilizing plant-based synthesis methods, reducing reliance on synthetic chemicals, and promoting biodegradable materials for industrial and biomedical use. By addressing challenges related to nanoparticle agglomeration, stability, and toxicity, this study contributes to eco-friendly material innovations, fostering a more sustainable and green future.

## 2. Results and Discussion

**Figure 1:**
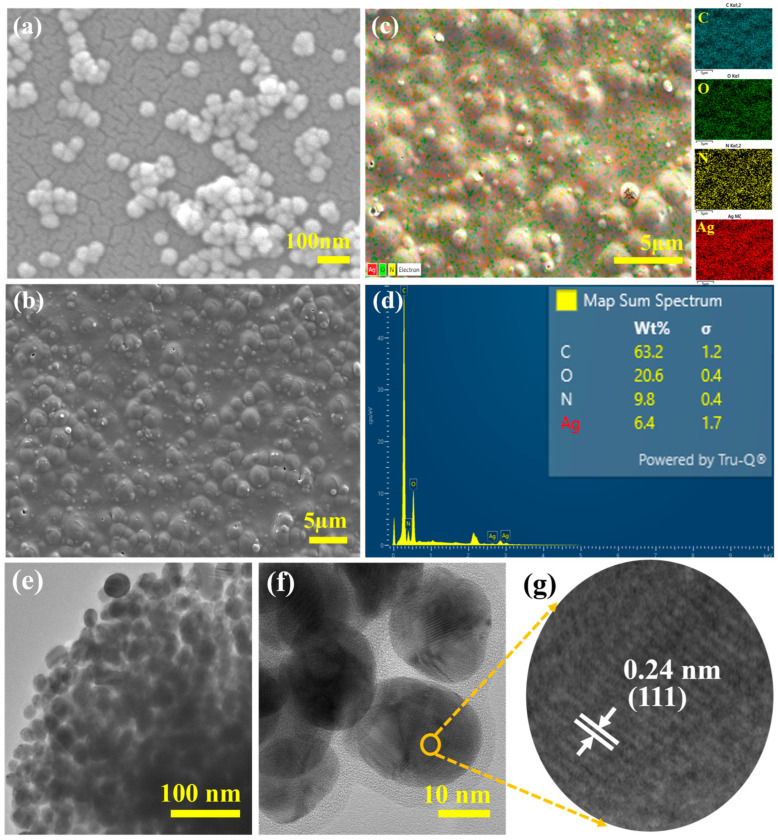
(**a**) SEM images of AgNPs, (**b**) SEM EDX mapping and (**c**) SEM image of the surface of the film-AgNP composite, (**d**) EDX spectra of the film, (**e**) HR-TEM image of AgNPs (low magnification), (**f**) HR-TEM image of AgNPs (higher magnification), and (**g**) image showing the lattice spacings within the crystals of the AgNPs.

Compared with conventional chemical methods, the synthesis of nanoparticles from plant sources serves as a natural, renewable, eco-friendly, and cost-effective mechanism, helping divert the dependence on synthetic and costly techniques. As a result, the effectiveness and continuity of plant-based synthesis are improving. The BC extract facilitates the formation of Ag nanoparticles. The compounds in the extract serve as reducing agents that aid in converting silver ions to silver atoms. The different phytochemicals, such as catechol, catechin, pyrogallol, gallic acid, and paashaanolactone, present in the BC effectively act as capping and stabilizing agents, thereby directing the morphology and size of the synthesized silver nanoparticles [10,19]. The aqueous medium used in the synthesis technique favorably aids in the complete dissociation of AgNO_3_ into ions and the release of ionic components from the extract, which enhances electrostatic interactions, contributing to the overall redox process involved in the reduction of Ag^+^ ions into Ag atoms. The surface plasmon resonance band of AgNPs usually appears between 300 and 700 nm, confirming their formation. The possibility of obtaining other water-soluble bioactive compounds, such as phenolic acids, flavonoids, alkaloids, some carbohydrates, and amino acids, in BC collectively contributes to reducing Ag^+^ ions into Ag nanoparticles. Figure 1a shows uniformly distributed Ag nanoparticles with an average diameter of approximately 40 nm. The particle size distribution was calculated via ImageJ 1.x (Figure S1). Transmission electron microscopy also confirmed the formation of spherical Ag nanoparticles (Figure 1e–g). The non-aggregated Ag nanoparticles further proved that the phytochemicals in the extract serve as potent capping agents. The high-resolution TEM results revealed that the outer side of the particles has less density, allowing light penetration with species from the plant extract attached to the particle surfaces. The high-resolution TEM images (Figure 1f) clearly show the core-shell nanostructure of the silver nanoparticles. The core is silver, and the shell consists of organic moieties from the extract. The organic shell with plant-derived compounds contributes significantly to interactions with film molecules during film formation. This creates strong bonding interactions between the AgNPs and the film, thereby enhancing the chemical and physical properties of the film for its intended application. The crystal lattice spacing (Figure 1g) is 0.24 nm, corresponding to the (111) plane, which agrees with the literature results of approximately 0.23 nm [20]. These findings confirmed that plant extracts can be used to synthesize AgNPs successfully.

The Ag nanoparticles were decorated on chitosan (CS), zein (Z), and CS-Z-BC thin films. The presence of zein in the synthesis process primarily contributed to film formation in the presence of chitosan, the main component of the composite, and other solvents, such as acetic acid, glycerol, and ethanol. The hydrophobic medium of synthesis was suitable for dissolving all the precursors and the crude extract of BC. After drying at 40 °C for 12 h, a brown film formed from the CS-Z-BC400-AgNPs, which contained AgNPs embedded film. The one-pot combination of all the precursor solutions during the synthesis process with the AgNPs resulted in a film with the AgNPs enclosed within the film, as shown in the FESEM images (Figure 1b). All the films were thin with uniform thicknesses ranging from 0.19 mm–0.20 mm ± 0.02 mm, with no significant cracking but some level of roughness observed, indicating adequate flexibility. This uneven surface morphology is due to the uneven drying of hydrophobic zein and hydrophilic chitosan, where zein can aggregate to decrease its exposure to the external environment, leading to different phases forming (Figure 1b). Collectively, the zein, chitosan, BC, and AgNPs aided in film formation. The EDX mapping shows the uniform distribution of silver nanoparticles over the thin film (Figure 1c). The elemental mapping shows that 6.4% of the weight of the silver nanoparticles, along with 63.2% for carbon, 20.6% for oxygen, 9.8% for nitrogen, and 6.4% for silver, are present in the film (Figure 1d). Carbon, oxygen, and nitrogen are present in chitosan, zein, and plant extracts.

**Figure 2:**
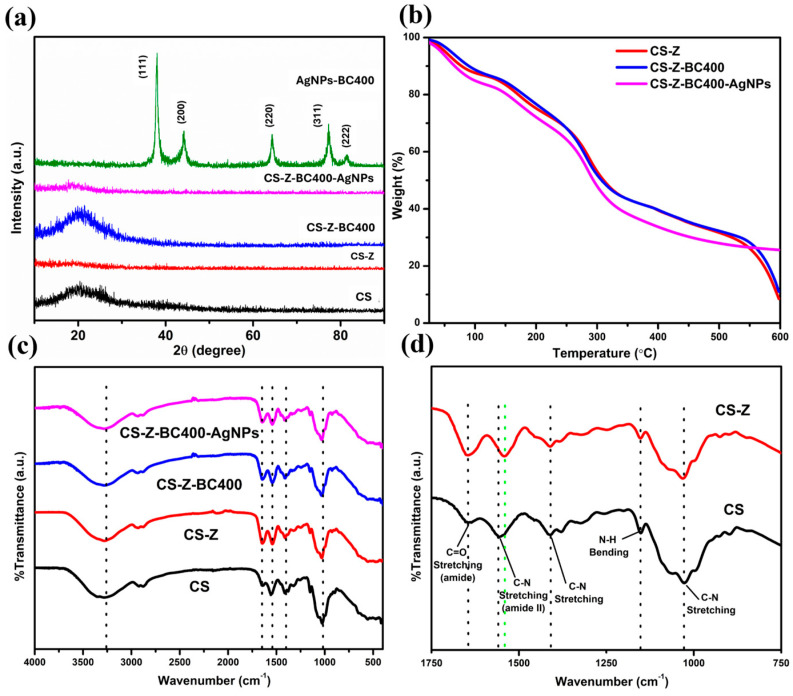
(**a**) XRD, (**b**) TG, (**c**,**d**) FTIR of CS, CS-Z, CS-Z-BC400, and CS-Z-BC400-Ag NPs.

The structure and crystallinity of the CS-Z-BC AgNPS film were investigated via X-ray diffraction (XRD) (Figure 2a). The XRD pattern obtained from AgNPs showed five distinct peaks located at 37.99°, 43.98°, 64.51°, 77.22°, and 81.32°, corresponding to the (111), (200), (220), (311), and (222) lattice planes of Ag metal, respectively (PDF# 04-002-1347), as shown in the supporting document (Figure S2). The XRD results revealed the formation of metallic Ag nanoparticles with a cubic crystal structure and Fm-3m (225) space group. The crystallite size of the silver nanoparticles calculated from the Scherrer equation was 15.5 nm. The one-pot combination of solutions during the synthesis process with the AgNPs resulted in a film with the AgNPs embedded within it. The XRD pattern of CS-Z-BC400-Ag does not have distinct diffraction peaks, as the Ag nanoparticles were embedded into the film. As expected, the CS, CS-Z, and CS-Z-BC400 samples presented only broad peaks at a Bragg angle of 20°, which can be attributed to the presence of carbon.

TGA analysis provided insight into the thermal stability of the synthesized film. The TGA thermogram and its derivative DTG curves (Figure 2b and Figure S3) obtained for the sample show decomposition in different steps with corresponding weight losses as the temperature increases to 600 °C, confirming the presence of different components of the synthesized material. The decrease in weight of approximately 15% from 25 °C to approximately 110 °C is characteristic of the loss of water and loosely bound low-molecular-mass volatile compounds from the sample. From the TGA derivative weight percent plot, three endothermic processes were observed at approximate temperature ranges of 50–110 °C, 140–240 °C, and 240–370 °C. All the thermograms exhibited the same temperature profile, with the trend in the Ag-decorated material showing a slightly lower degradation rate and stable weight above 600 °C. The constant weight of the CS-Z-BC400-AgNPs sample after approximately 70% weight loss confirms the presence of AgNPs, which do not degrade. The CS-Z, and CS-Z-BC400, samples completely decomposed at approximately 600 °C. The second degradation step between 150 °C and approximately 240 °C could be due to the removal of glycerol, which has been reported to have a boiling point of 198 °C [21], chitosan [17], and hemicellulose [22] with a corresponding total weight loss of approximately 20%. There is the possibility of removing fatty acids that degrade at this same temperature. The third degradation step between 240 °C and approximately 330 °C could be due to zein degradation, which usually occurs at 270 °C to 415 °C [23] with a weight loss of approximately 30%. The gradual degradation step above 350 °C can be attributed to the decomposition of zein in the sample. From the TGA results, it can be concluded that the synthesized material is thermally stable when used at room temperature and relatively high temperatures to a maximum of approximately 200 °C, beyond which vital components may be lost because of the high degradation rate. The addition of chitosan and zein further improved the thermal stability of the material since they decompose at relatively higher temperatures than ambient temperature, as only approximately 30% of the weight was lost at approximately 200 °C, most of which could be moisture, with the most significant weight loss, approximately 50%, occurring above 250 °C. The residue formed after calcination is shown in the Supporting Information (Figure S4).

Fourier transform infrared (FTIR) analysis of the films (Figure 2c,d) revealed vibrational bands, confirming the functional groups expected in the synthesized samples. The FTIR spectra obtained from 4000 to 500 cm^−1^ showed strong similarity in intensity and band locations, with slight peak shifting occurring after the addition of zein to the chitosan. The 1539 cm^−1^ band in the spectrum of chitosan corresponds to the N-H bending (amide II) vibration shifted to 1561 cm^−1^ in the composite containing zein. This shows a strong interaction between the chitosan and the zein. The increase in the intensity of the 1653 cm^−1^ peak in all the composites compared with that of the chitosan shows a strong interaction between zein and chitosan through strong hydrogen bonds and intermolecular interactions [24]. No significant peak shifting was observed for the different samples containing zein. The absorption band observed at approximately 1653 cm^−1^ can be attributed to the C=O stretching vibration, possibly from the amide group from zein. The samples exhibited a significant absorption band at 1037 cm^−1^, characteristic of the C-N stretching vibration. O-H and N-H stretching vibration bands, which usually occur at approximately 3200 cm^−1^ and 3300 cm^−1^ [25] respectively, were not seen because a broad band was observed between 3000 cm^−1^ and 3700 cm^−1^ for all the samples. The peak at 2930 cm^−1^ is characteristic of C-H stretching vibrations, with a close peak at 2880 cm^−1^, which can be attributed to the symmetric C-H stretching vibrations of CH_3_ groups. The 1160 cm^−1^ and 1415 cm^−1^ peaks refer to N-H bending vibrations and C-N stretching vibrations, respectively, whereas the 1551 cm^−1^ band refers to C-N stretching vibrations of an amide group [24].

**Figure 3:**
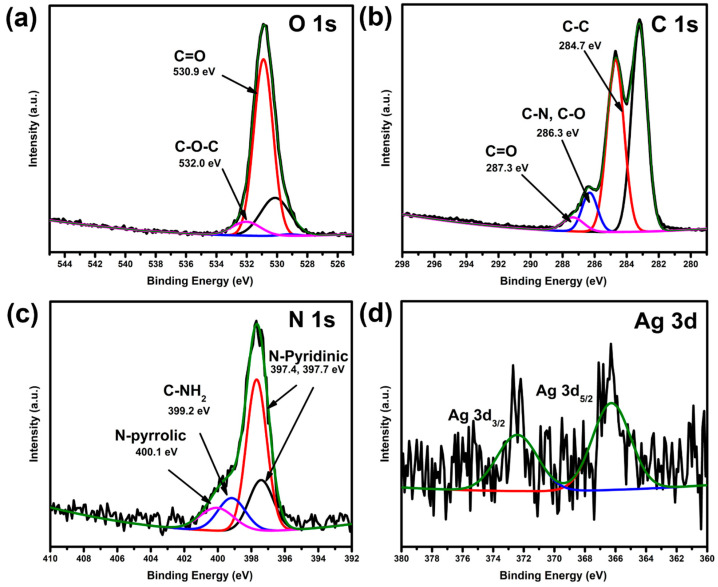
XPS spectra of (**a**) O 1s, (**b**) C 1s, (**c**) N 1s, and (**d**) Ag 3d of the composite polymer films.

XPS analysis assisted in studying the chemical composition and interaction of the AgNPs with the polymer film, contributing to the enhanced activity of the film. The survey spectra obtained from X-ray photoelectron spectroscopic analysis revealed the presence of C 1s, N 1s, and O 1s peaks, with a low peak intensity of the Ag 3d peak due to the controlled amount used in the composite (Figure S5). The O 1s spectrum (Figure 3a) showed a peak at 530.9 eV, corresponding to C=O bonding in the film. The peak at 532.0 eV can be attributed to C-O-C bond interactions. The peak observed in the O 1s spectrum with a binding energy of 529.2 eV is usually attributed to the lattice oxygen in the metal oxide [26,27] and the crystal structures can be attributed to the interaction of the AgNPs with the oxygen from the polymer. This shows the strong interaction of the AgNPs with the polymer film. The carbon 1s spectrum (Figure 3b) revealed binding energies at 284.7, 286.3, and 287.3 eV, which can be attributed to C-C, C-N, or C-O and C=O, respectively. The binding energy observed at 283.2 eV can be attributed to graphitic carbon or carbon interactions with AgNPs, as C bonded to metals has a binding energy of approximately 282 eV [28] or C=C bonds [22,29]. This possible Ag-C interaction contributes to slightly splitting the C 1s peak between 283 and 284 eV. The nitrogen 1s spectrum (Figure 2c) shows binding energies at 397.4, 399.2, and 400.1 eV, indicating the presence of N in pyridinic, amide, and pyrrolic chemical environments. This confirms that the film contains N-based monomeric species. The deconvoluted silver 3d spectrum (Figure 3d) shows peaks with binding energies of 366.3 eV and 372.4 eV corresponding to Ag 3d_5/2_ and Ag 3d_3/2_, respectively can be attributed to metallic Ag (Ag°) with zero oxidation state. The spectra yielded an area ratio of 2.9:2, close to the expected area ratio of 3:2 for the 3d_5/2_ and 3d3_3/2_ orbitals, with a binding energy separation of 6.1 eV. The slight shift in the binding energy of the Ag 3d_5/2_ peak is usually expected at 368 eV, and the Ag 3d_3/2_ at 374 eV [29] can be attributed to the bonding interactions between the AgNPs and the species around the AgNPs.

**Figure 4:**
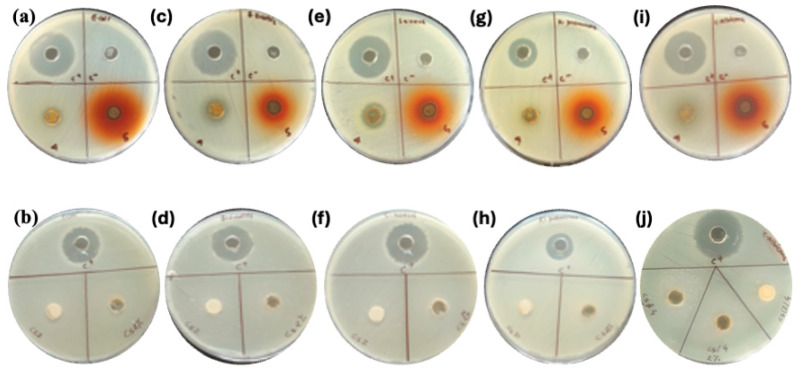
Antibacterial activity of different films against various bacterial strains. *E. coli* (**a**,**b**), *B. subtilis* (**c**,**d**), *S. aureus* (**e**,**f**), *K. pneumoniae* (**g**,**h**) and *C. albicans* (**i**,**j**). 4 and 5 represent CS-Z-BC400 and CS-Z-BC400 AgNPs respectively.

The antimicrobial effectiveness of CS-Z composites, particularly when incorporated with BC extract and silver nanoparticles (AgNPs), was evaluated against a range of microbial strains, including both Gram-negative and Gram-positive bacteria, as well as fungi as shown in Figure 4 and Table 1.

The control sample (CS-Z) exhibited no detectable inhibition (ND) across all tested strains. In contrast, adding BC extract (CS-Z-BC400) resulted in inhibition zones ranging from 12 mm to 22 mm. The largest inhibition zone was recorded against *C. albicans* (22 mm), indicating a comparatively higher activity against this fungal strain. Upon incorporation of AgNPs (CS-Z-BC-AgNPs), the antibacterial activity was further enhanced for most strains. Inhibition zones for *E. coli*, *B. subtilis*, and *S. aureus* increased to 18 mm, 17 mm, and 16 mm, respectively, compared to 12 mm, 14 mm, and 13 mm with CS-Z-BC400, suggesting a possible additive or synergistic interaction between BC extract and AgNPs. However, the antifungal activity against *C. albicans* decreased slightly from 22 mm to 17 mm, indicating that including AgNPs may alter the overall efficacy against fungal organisms. These findings highlight that the combination of BC and silver nanoparticles enhances the antimicrobial performance of CS-Z composites, particularly in bacterial inhibition.

AgNPs, as reported, exhibit antimicrobial activity through the cell wall and membrane disruption, silver ion release which tends to bind cellular components such as proteins, DNA, enzymes, and consequently disrupting their functions, inhibiting biofilm formation, and disruption, induction of production of reactive oxygen species triggering oxidative stress and cell damage, interaction with ribosomes and other cellular materials thereby inhibiting protein synthesis required for cell growth and cell division [30,31]. In addition to the mechanism by which AgNPs are in antimicrobial activity, the formation of a coating by organic moieties through the green synthesis of the AgNPs in our study possibly provided an interface for easy adherence of the AgNPs to the biological cell structures. The enhancement of the antimicrobial activity of our material can also be attributed to the contribution of the thin film coating around the AgNPs which possibly enhanced the interaction of the AgNPs with cell structures creating an interface for easy attachment.

The biodegradability of the samples was assessed based on weight loss over 28 days, with the results presented in Table 2. Among the tested samples, CS-Z exhibited the highest weight loss of 55.21%, indicating its high susceptibility to microbial degradation and low resistance to environmental breakdown. This rapid degradation highlights the inherent biodegradability of chitosan-zein films but also suggests limitations in their long-term usability. In contrast, the CS-Z-BC400-AgNPs composite film demonstrated the lowest weight loss, at 40.71%, over the same period, suggesting moderate biodegradability. The reduced degradation rate of this sample can be attributed to the incorporation of BC extract and silver nanoparticles, which significantly enhance the film’s antimicrobial efficacy. By inhibiting microbial growth on the film’s surface, these bioactive additives slow down the microbial-driven degradation process in the soil, as further confirmed by microbial testing.

These findings suggest that the lifespan of chitosan-zein films can be effectively prolonged by incorporating plant extracts and AgNPs. While maintaining essential biodegradable properties, these modifications enhance film stability, making them more suitable for applications requiring both antimicrobial protection and extended durability.

## 3. Materials and Methods

### 3.1. Materials

Zein protein (grade Z3625, 22–24 kDa) and 75% deacylated chitosan-448877, having a medium molecular weight (340 g/mole), were purchased from Sigma-Aldrich (St. Louis, USA). 99.9% pure silver nitrate (AgNO_3_) was purchased from Fisher Scientific (Brussel, Belgium). Ethanol and acetic acid were purchased from Qualigens. Deionized water was used to extract and prepare the solution.

### 3.2. Preparation of BC Plant Extract

Fresh leaves and rhizomes of BC plants were collected from the upper part of the Dolpa district, Nepal, and thoroughly washed with distilled water to remove dirt and impurities. The plant material was then dried under shade to retain its bioactive compounds. The dried plant material was ground into a fine powder via a grinder. 5 g of the powdered plant material was mixed with 100 mL of distilled water and heated for 40 min with a magnetic stirrer at 60 °C. This helps with the extraction of bioactive compounds. The mixture was then cooled and filtered through Whatman filter paper to obtain a clear extract.

### 3.3. Synthesis of Silver Nanoparticles

1 mM silver nitrate (AgNO_3_) solution was prepared by dissolving the required amount of AgNO_3_ in distilled water. This solution served as the silver precursor in the synthesis process. The aqueous plant extract was slowly added to the silver nitrate solution at a 1:9 ratio under constant stirring at laboratory temperature 23 °C and kept in the dark, resulting in a color change in the solution from pale yellow to dark brown, indicating the formation of Ag nanoparticles.

### 3.4. Synthesis of Chitosan–Zein Films Decorated with AgNPS

A 1% (*w*/*v*) zein solution was prepared by dissolving 1 g of zein protein in 100 mL of 70% (*v*/*v*) ethanol at room temperature under constant stirring. Similarly, a 2% (*w*/*v*) chitosan solution was prepared by dissolving 2 g of chitosan in 100 mL of 1% (*v*/*v*) acetic acid, followed by the addition of 0.6 g of glycerol. This mixture was stirred continuously for 4 h at 80 °C to ensure complete dissolution. Additionally, 400 mg of crude BC extract was dissolved in 99.9% ethanol, 17 mg of silver nanoparticles (AgNPs) was dispersed in 1 mL of ethanol, subjected to sonication for proper dispersion, and added to the mixture.

The zein, chitosan, and solution of BC containing 400 mg of it, along with the AgNPs dispersion, were centrifuged at 8000 rpm for 10 min to remove impurities. The purified solutions were then combined and stirred for 2 h at room temperature to achieve homogeneity. To fabricate the films, 35 mL of each solution (individual and combined) was poured onto Petri dishes (25 cm × 25 cm) to ensure uniform thickness. The films were dried in a ventilated oven at 40 °C for 12 h, then carefully peeled off. The dried films were labeled as CS, CS-Z, CS-Z-BC400, and CS-Z-BC400 AgNPs and stored in a dry environment, wrapped in aluminum foil to prevent moisture absorption and light exposure before further testing.

### 3.5. Characterization

#### 3.5.1. Morphology and Elemental Mapping

Field emission scanning electron microscopy (FESEM, JEOL, JSM-IT800) from Peabody, MA, USA, is used to study the surface structure and shape of the synthesized film. The elemental mapping was performed to identify the elements present using energy dispersive X-ray spectroscopy (EDS) from Oxford Instruments, Concord, MA, USA. The EDS is coupled with the FESEM. The structural analysis, morphology, and crystalline nature of the AgNPs were studied via transmission electron microscopy (TEM, Model JEOL JEM-2100plus), Peabody, MA, USA, and the interplanar distances were measured via ImageJ software.

#### 3.5.2. Crystallinity

The crystallinity and purity of the samples were checked on an X-ray diffractometer via a Rigaku Miniflex 600 (source: Cu Kα, wavelength: 1.5406 Å, 2θ: 10–90, step: 0.02, and scan rate: 2°/min) purchased from Woodlands, TX, USA.

#### 3.5.3. Thermal Properties and Stability Studies

The thermal properties of the fabricated films were investigated via thermogravimetric analysis (TGA 5500, TA Instruments), New Castle, DE, USA. Approximately 13 mg of the film was placed in a platinum sample holder and heated at a ramp rate of 10 °C/min from 25 to 600 °C.

#### 3.5.4. FTIR Spectroscopic Studies

The Fourier transform infrared (FTIR) spectra of the samples were measured with an IRTracer-100, from Shimadzu Scientific Instruments, Columbia, MD, USA.

#### 3.5.5. XPS Spectroscopic Analysis

Further elemental analysis and chemical bond interactions were performed using X-ray photoelectron spectroscopy (XPS, Escalab Xi+), Thermo Fisher Scientific, Waltham, MA, USA.

### 3.6. Antimicrobial Test

The antimicrobial activity of the samples was assessed using the well diffusion method. Liquid Broth (LB) medium was prepared by dissolving 13 g of LB powder in 1 L of distilled water, followed by sterilization at 121 °C for 25 min under 15 psi pressure. After cooling to 40–50 °C, the sterile medium was transferred into 15 mL Falcon tubes (5 mL each) for bacterial seed culture preparation. Standard ATCC bacterial and fungal strains were inoculated into the LB broth and incubated at 37 °C for 24 h.

#### 3.6.1. Inoculum Preparation and Plate Inoculation

A 150 µL aliquot of the freshly prepared bacterial culture (1.5 × 10^8^ CFU/mL) was uniformly spread onto Mueller–Hinton Agar (MHA) plates using sterile cotton swabs. MHA plates were prepared by dissolving 39 g of MHA powder (Sisco Research Laboratories Pvt. Ltd., Mumbai, India) in 1 L of distilled water, followed by autoclaving at 121 °C for 25 min under 15 psi pressure. The sterilized agar was cooled to 40–50 °C, poured into sterile Petri dishes (25 mL per dish), and solidified.

#### 3.6.2. Disc Accommodation

To accommodate three discs per sample, wells (9 mm diameter, 3 mm depth) were gently made in the agar surface using the back of a sterile pipette tip. Two discs were placed inside the wells for better contact and diffusion, while the third was positioned on the agar surface. Standard kanamycin solution (5 mg/mL, 10 µL) was used as a positive control.

#### 3.6.3. Incubation

The plates were incubated at 37 °C for 24 h. The zones of inhibition were measured to evaluate antimicrobial activity. Chitosan–zein (CS-Z) film was used as the control sample. The microbial strains used were *Escherichia coli* (ATCC 8739), *B. subtilis* (ATCC 6051), *S. aureus* (ATCC6538P), *K. pneumoniae* (ATCC 700603), and *C. albicans* (ATCC 2091), all obtained from certified microbial culture suppliers.

### 3.7. Biodegradation Test

The biodegradability of films was assessed using a composting test, as described in the literature with slight modification [25]. In this method, films were cut into standardized 2 × 5 cm samples and buried 6 inches deep in soil sourced from a local nursery for 28 days. To simulate a consistent composting environment, the soil was moistened by spraying water in the morning and kept at room temperature. After each week, for four weeks, the film samples were extracted, washed with distilled water, dried at 105 °C, and weighed to determine weight loss, indicative of their biodegradability. While this controlled test provides a baseline measure of biodegradability, it’s important to consider that real-world conditions, influenced by variables like soil pH, microbial activity, and temperature, can yield different results. The pure chitosan-zein sample served as a control for comparison. The weight loss of the sample is then calculated by the following formula.

% Weight loss = (Initial wt. − Final wt.)/(Initial wt.) × 100

## 4. Conclusions

In this study, we successfully developed chitosan–zein films incorporated with silver nanoparticles synthesized via a green method using *Bergenia ciliata* extract as a natural reducing and stabilizing agent. This eco-friendly approach eliminates the need for hazardous reducing agents such as sodium borohydride or lithium butyl borohydride, aligning with green chemistry principles. The resulting films demonstrated potent antimicrobial activity against Gram-positive and Gram-negative bacteria, enhanced by the synergistic effect of chitosan, zein, BC extract, and AgNPs. Additionally, the films showed controlled biodegradability and improved structural durability due to reduced microbial degradation. The thermogravimetric analysis confirmed the thermal stability of the CS-Z-BC400 AgNPs composite, withstanding temperatures up to 200 °C, making it suitable for applications at room and moderately elevated temperatures. Altogether, these results suggest that the developed material is a promising, sustainable alternative for antimicrobial and biodegradable applications in biomedical, food packaging, and related industries.

## Figures and Tables

**Table 1:** In vitro antimicrobial activity of the silver nanoparticles decorated chitosan-zein-BC film.

Strain	Reference Culture	Type	C+ Kanamycin	* Effectiveness (Inhibition Zone, mm)		
				CS-Z Control	CS-Z-BC 400	CS-Z-BC-AgNPs
*Escherichia coli*	ATCC8739	Gram −ve	26	ND	12	18
*Klebseilla pneumoniae*	ATCC 700603	Gram −ve	18	ND	14	15
*Bacillus subtilis*	ATCC 6051	Gram +ve	25	ND	14	17
*Staphylococcus aureus*	ATCC6538P	Gram +ve	25	ND	13	16
*Candida albicans*	ATCC2091	Fungi	27	ND	22	17

* The zone of inhibition (ZOI) has been tabulated in mm. Kanamycin antibiotic in the concentration of 5 mg/mL has been used as a positive control (c+).

**Table 2:** Biodegradability of different samples over 28 days and corresponding weight loss (%).

Day/Sample	CS	CS-Z	CS-Z-BC400	CS-Z-BC400-AgNPs
1	0.111	0.096	0.127	0.140
7	0.094	0.078	0.125	0.137
14	0.0631	0.074	0.087	0.1023
21	0.057	0.047	0.072	0.084
28	0.053	0.043	0.071	0.083
Wt. Loss %	52.45%	55.21%	44.09%	40.71%

## Data Availability

Data are contained within the article and in the electronic Supplementary Information.

## Supplementary Materials

The following supporting information can be downloaded at: https://www.mdpi.com/article/10.3390/molecules30112311/s1, Figure S1: Particle size distribution of synthesized silver nanoparticles (AgNPs) measured using ImageJ.; Figure S2: Crystalline size determination of AgNPs calculated using the full width at half maximum (FWHM) of the maximum peak from XRD pattern and Debye Scherrer equation.; Figure S3: Differential thermogravimetric spectra of CS, CS-Z, CS-Z-BC and CS-Z-BC-Ag.; Figure S4: Film before and after TGA analysis.; Figure S5: XPS survey spectrum of composite polymer films.; Table S1: Calculation of crystalline size of synthesized AgNPs.; Table S2: Tableted summary of XRD peaks of AgNPs and their standard (PDF# 04-002-1347).

## Abbreviations

The following abbreviations are used in this manuscript:

AgNPs	Silver nanoparticles
CS	Chitosan
BC	*Bergenia ciliata*
Z	Zein
FESEM	Field emission scanning electron microscopy
TGA	Thermogravimetric analysis
LB	Liquid broth
HRTEM	High-resolution transmission electron microscopy
EDX	Energy-Dispersive X-ray Spectroscopy
XRD	X-ray Diffraction Spectroscopy
FTIR	Fourier transport infrared
ATCC	American Type Culture Collection
ZOI	Zone of inhibition
