# Supplementary material for: Chitosan–Zein Films Decorated with Green Synthesized Silver Nanoparticles Using Bergenia ciliata Extract
Source: Molecules. 2025 May 24;30(11):2311. doi: 10.3390/molecules30112311 (PMC12156224; doi:10.3390/molecules30112311)
Supplement: Supplementary file 1 [file molecules-30-02311-s001.zip › molecules-3585443-supplementary.pdf]

Electronic Supplementary Information

Chitosan–Zein Films Decorated with Green Synthesized Silver Nanoparticles using Bergenia ciliata Extract

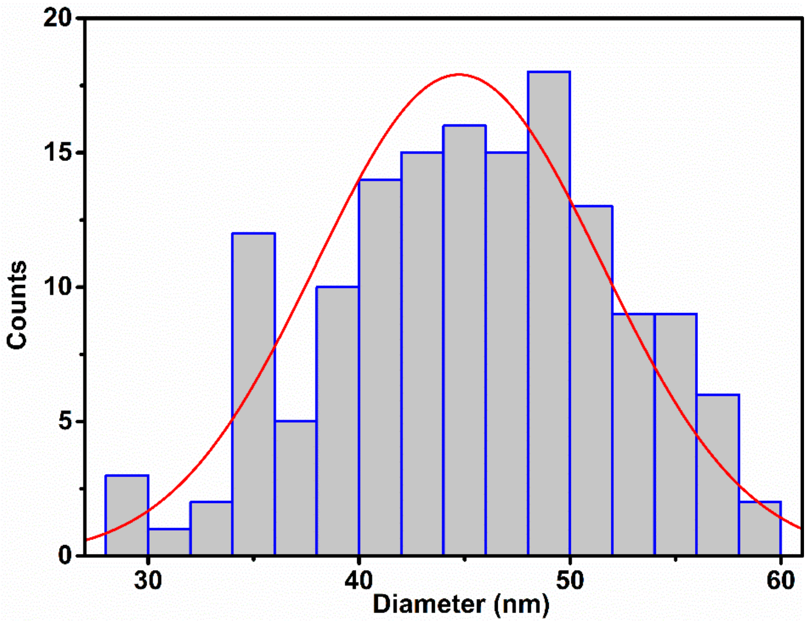

Figure S1. Particle size distribution of synthesized silver nanoparticles (AgNPs) measured using ImageJ.

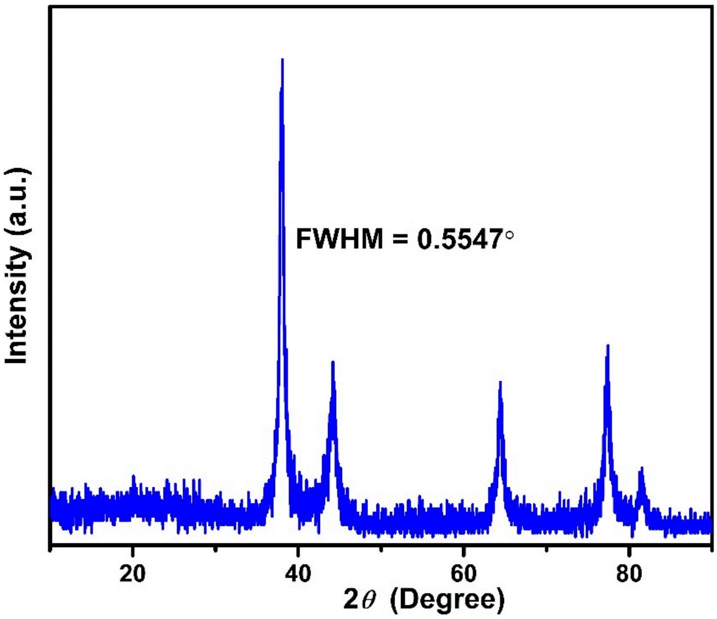

Figure S2. XRD pattern of AgNPs. Crystalline size determination of AgNPs calculated using the full width at half maximum (FWHM) of the maximum peak from XRD pattern and Debye Scherrer equation;

$$D = \frac{K\lambda}{\beta Cos\theta}$$

Where: D is the mean crystallite size
λ is the X-ray wavelength
K is the shape factor (typically around 0.9)
β is the line broadening at half the maximum intensity (FWHM), in radians

Table S1: Calculation of crystalline size of synthesized Ag NPs.

Sample	FWHM (Degree)	FWHM (Radians)	Bragg's angle (Degree)	Crystalline size (D)
AgNPs	0.5574	0.009728	37.99	15.51053

Table S2: Tableted summary of XRD peaks of AgNPs and their standard (PDF# 04-002-1347).

No.	*Peak position AgNPs* (2*θ*)	*Standard peak position* (2*θ*)	*h*	*k*	*l*
1.	37.99°	37.980°	1	1	1
2.	43.98°	44.141°	2	0	0
3.	64.51°	64.198°	2	2	0
4.	77.22°	77.086°	3	1	1
5.	81.32°	81.206°	2	2	2

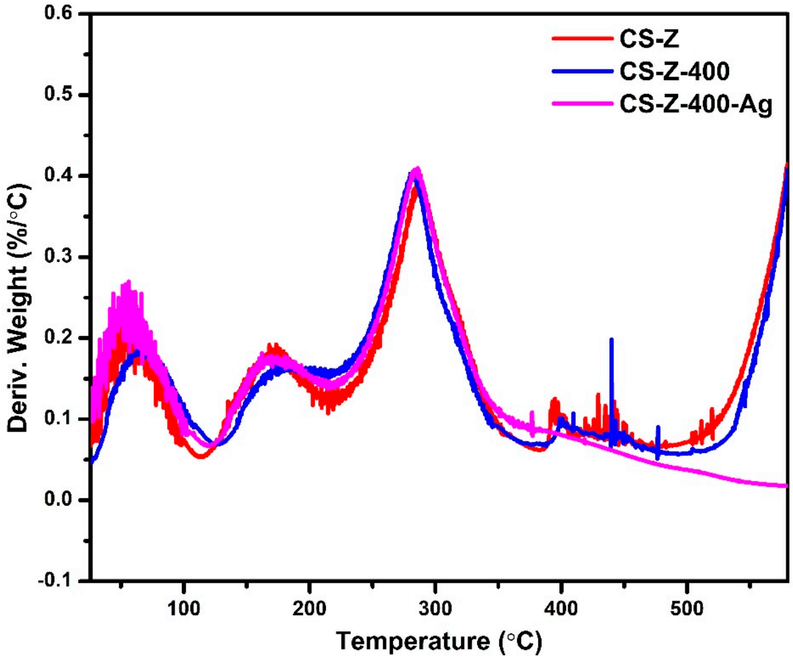

Figure S3. Differential thermogravimeteric specta of CS, CS-Z, CS-Z-BC, and CS-Z-BC-Ag.

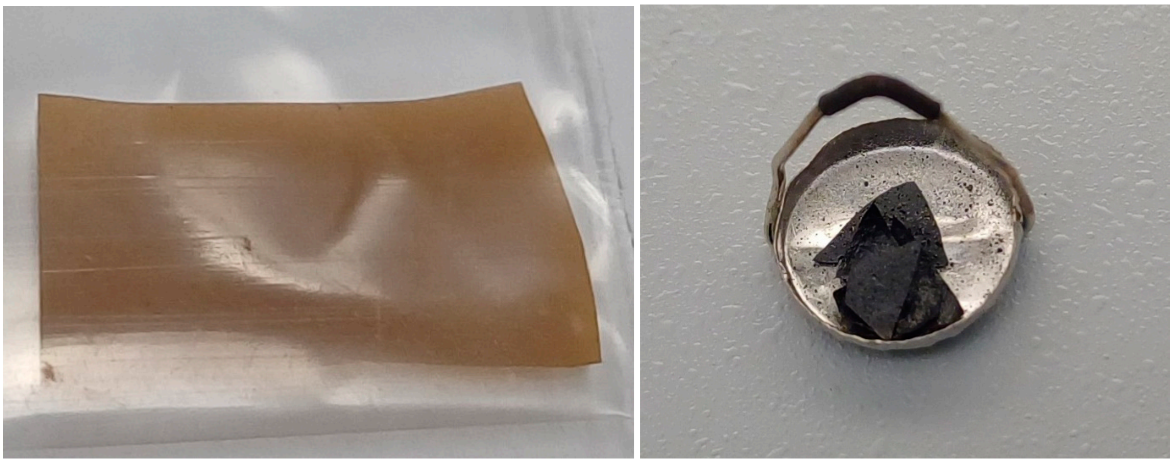

Figure S4. Film before and after TGA analysis.

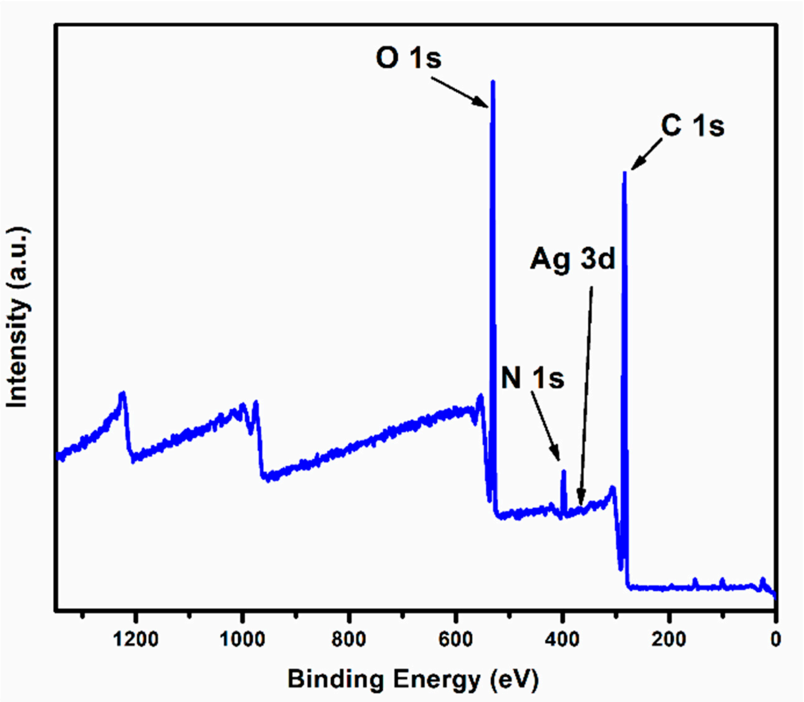

Figure S5: XPS survey spectrum of composite polymer films.
